# NET formation can occur independently of RIPK3 and MLKL signaling

**DOI:** 10.1002/eji.201545615

**Published:** 2015-12-02

**Authors:** Poorya Amini, Darko Stojkov, Xiaoliang Wang, Simone Wicki, Thomas Kaufmann, Wendy Wei‐Lynn Wong, Hans‐Uwe Simon, Shida Yousefi

**Affiliations:** ^1^ Institute of PharmacologyUniversity of BernBernSwitzerland; ^2^ Institute of Experimental ImmunologyUniversity of ZurichZurichSwitzerland

**Keywords:** MLKL, Necroptosis, NET formation, NETosis, Neutrophils, RIPK

## Abstract

The importance of neutrophil extracellular traps (NETs) in innate immunity is well established but the molecular mechanisms responsible for their formation are still a matter of scientific dispute. Here, we aim to characterize a possible role of the receptor‐interacting protein kinase 3 (RIPK3) and the mixed lineage kinase domain‐like (MLKL) signaling pathway, which are known to cause necroptosis, in NET formation. Using genetic and pharmacological approaches, we investigated whether this programmed form of necrosis is a prerequisite for NET formation. NETs have been defined as extracellular DNA scaffolds associated with the neutrophil granule protein elastase that are capable of killing bacteria. Neither *Ripk3*‐deficient mouse neutrophils nor human neutrophils in which MLKL had been pharmacologically inactivated, exhibited abnormalities in NET formation upon physiological activation or exposure to low concentrations of PMA. These data indicate that NET formation occurs independently of both RIPK3 and MLKL signaling.

## Introduction

Extracellular DNA traps are a part of the innate immune response and are seen with many infectious, allergic and autoimmune diseases. They can be generated by several different leukocytes, including neutrophils, eosinophils, basophils and monocytes, as well as mast cells 1, whereby the neutrophil extracellular traps (NETs) have been most frequently studied. Extracellular DNA traps can bind and kill bacteria 2 and fungi 3 in the extracellular space, but may also contribute to immunopathology 2 or even cause autoimmunity 4, 5. While most investigators agree that NETs contribute significantly to innate immunity, the molecular mechanisms responsible for their formation remain unclear and in dispute. Our focus is a simple question: Does the neutrophil need to die in order to provide the extracellular DNA scaffold characteristic for NETs or not?

Necroptosis is a newly discovered regulated cell death pathway that requires the functions of receptor‐interacting protein kinase 3 (RIPK3) and mixed lineage kinase domain‐like (MLKL) protein 6, 7, 8, 9. Genetic rescue studies using RIPK3 and MLKL knockout mice suggested that necroptosis triggers inflammation 6, 7, 8, 9. Interestingly, recent reports also indicate that RIPK3 may contribute to production of pro‐inflammatory cytokines independently of its necroptotic activity 10, 11.

The type of cell death postulated as being required for NET formation has been called “NETosis” 12, 13, 14. Since it has been suggested that a neutrophil nonapoptotic type of death is required for NET formation in inflammatory processes 2, 12, 13, 14, it seemed possible that the RIPK3‐MLKL signaling pathway might regulate this process. The aim of this study was to investigate whether NETosis is actually necroptosis and whether RIPK3 contributes to NET formation. Using genetically deficient mice and pharmacological approaches, we now report that NET formation is a RIPK3‐MLKL‐independent process.

## Results and discussion

### *Ripk3*‐deficient neutrophils are able to form functional NETs

**Figure 1:**
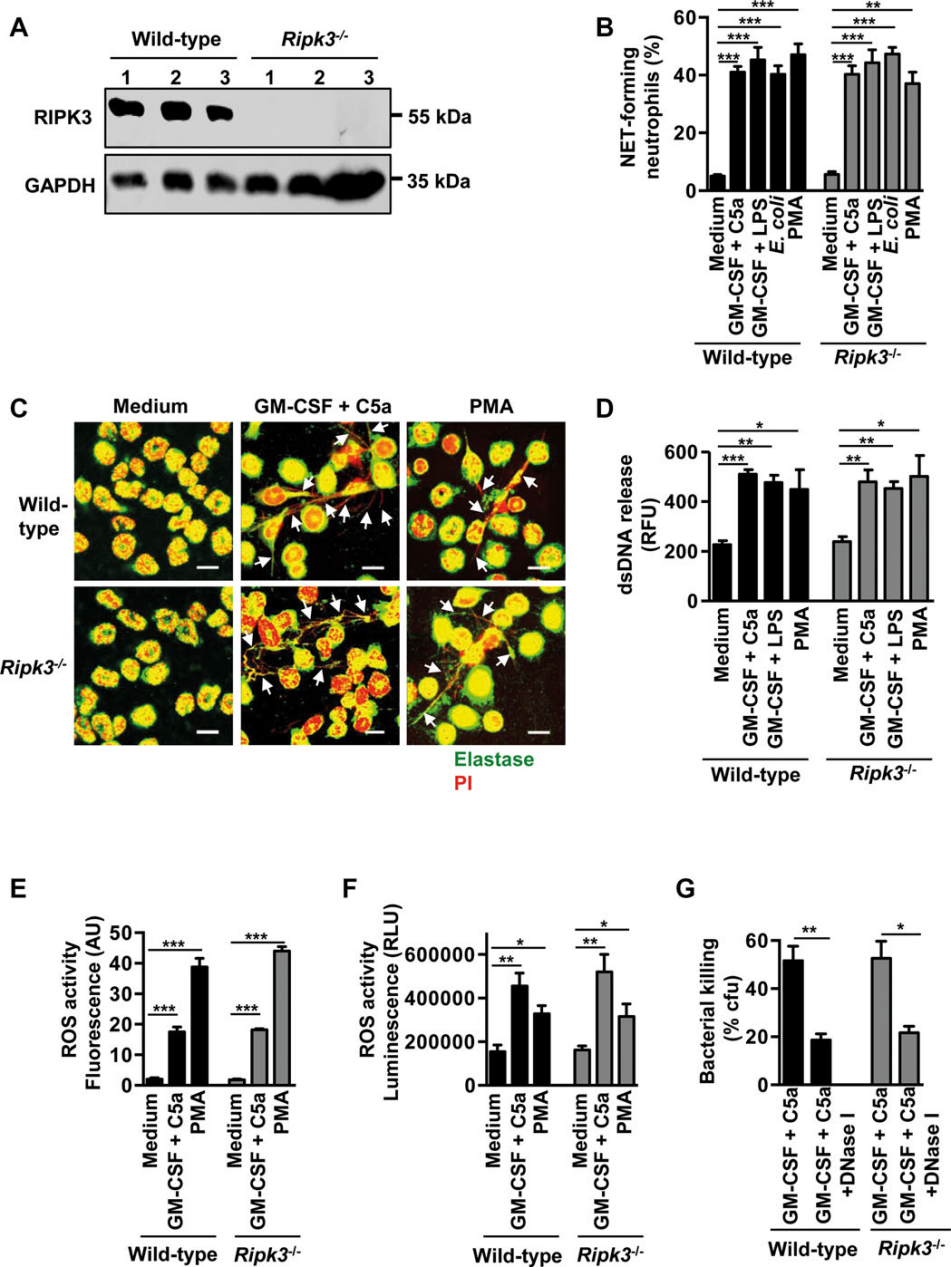
**The formation of mouse NETs is independent of RIPK3**. Mature mouse neutrophils were isolated from bone marrow of wild‐type and *Ripk3*‐deficient mice. (A) Immunoblotting. Bone marrow cells from wild‐type and *Ripk3*‐deficient mice were analyzed for RIPK3 protein expression. Three mice per genotype are shown. (B) Quantification of NET‐forming neutrophils by confocal microscopy. NET formation following short‐term stimulation (total 45 min) of mouse neutrophils with the indicated triggers. The number of NET‐forming neutrophils was determined by counting the DNA‐releasing cells in ten high power fields (Supporting Information Fig. 1A and Supporting Information Movie 1). No statistical differences were observed between wild‐type and *Ripk3*‐deficient cells (*n* = 3). (C) Representative microscopy. Co‐localization (arrows) of elastase (green) with released DNA (PI, red) assessed by confocal microscopy. Bars, 10 μM. (D) Quantification of dsDNA in supernatants of activated neutrophils using PicoGreen fluorescent dye. A significant difference in dsDNA release was detected between control and activated cells, but not between wild‐type and *Ripk3*‐deficient neutrophils (*n* = 3). (E) Total ROS activity assessed by DHR123 fluorescence and flow cytometry (*n* = 3). (F) Quantification of H_2_O_2_ production upon activation of neutrophils was performed using luminescent ROS‐Glo that measures H_2_O_2_ levels directly in cell culture. Again, ROS activity is increased in activated mouse neutrophils, but no statistical differences were observed between wild‐type and *Ripk3*‐deficient cells (*n* = 3). (G) Bacterial killing by colony formation unit (cfu) assay (*n* = 3). Mouse neutrophils exert antibacterial activity against *E. coli* that can be partially blocked by 100 U/mL DNase I. All data are shown as mean ± SEM of the indicated number of independent experiments. *p < 0.05; **p < 0.01; ***p < 0.001; one‐way ANOVA test.

To investigate whether NETosis is actually necroptosis, we stimulated *Ripk3*‐deficient GM‐CSF‐primed mouse neutrophils with C5a or LPS, or alternatively, in the absence of priming, with *E. coli* or phorbol‐12‐myristate‐13‐acetate (PMA), all known triggers of NET formation. *Ripk3*‐deficient neutrophils (Fig. 1A) were able to form NETs just as well as neutrophils from wild‐type mice (Fig. 1B and Supporting Information Fig. 1A) and showed no increased spontaneous cell death (Supporting Information Fig. 1B). However, as expected, *Ripk3*‐deficient neutrophils were less susceptible to undergoing necroptosis as compared to wild‐type neutrophils (Supporting Information Fig. 1B). In addition, the stimuli used to trigger NET formation did not induce cell death within a time period of 1 h (Supporting Information Fig. 1C and Movie 1). We established the formation of NETs by demonstrating the presence of extracellular DNA fibers associated with elastase (Fig. 1C).

To quantify the dsDNA released by activated neutrophils, we also collected the culture supernatants and measured the amount of released dsDNA using PicoGreen fluorescent dye 15. We observed no differences in dsDNA release between *Ripk3*‐deficient and wild‐type mouse neutrophils, but, as expected, there were statistically significant differences in dsDNA release between control and activated neutrophils derived from both wild‐type and *RIPK3‐*deficient mouse neutrophils (Fig. 1D). Moreover, *Ripk3*‐deficient neutrophils were able to generate the same amounts of reactive oxygen species (ROS), measured both by fluorescent and luminescent methods (Fig. 1E and F), which are known to be required for NET formation 16. In addition, *Ripk3*‐deficient neutrophils exhibited the same extracellular antibacterial killing activity as wild‐type neutrophils (Fig. 1G). Taken together, these data clearly demonstrate that NET formation by mouse neutrophils is independent of RIPK3.

### Pharmacological inhibition of MLKL does not prevent functional NET formation by human neutrophils

**Figure 2:**
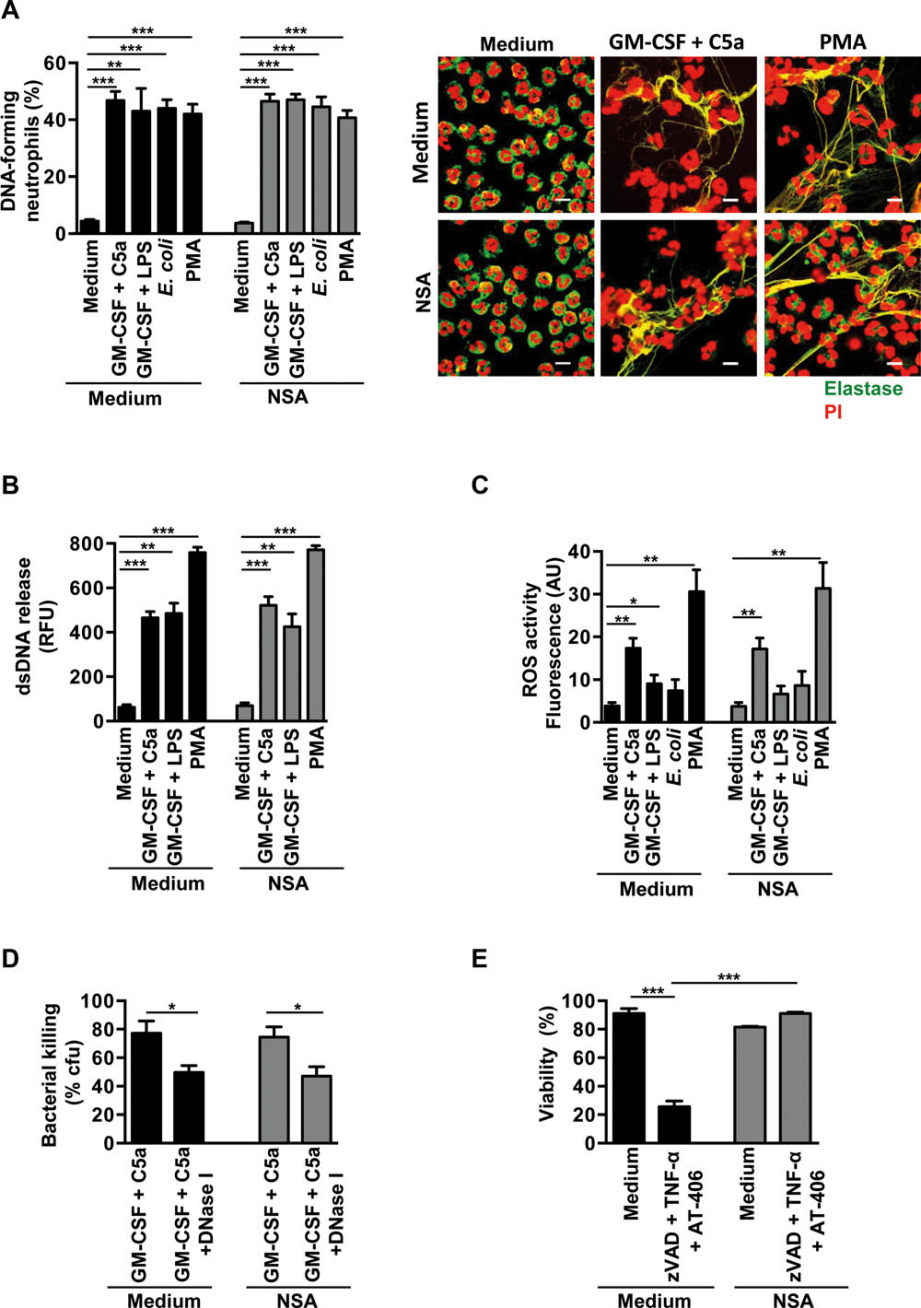
**The formation of human NETs occurs independently of MLKL signaling**. Human blood neutrophils were isolated from healthy donors by Ficoll‐Hypaque centrifugation. (A) Confocal microscopy. NET formation following short‐term stimulation (total 45 min) of human neutrophils with the indicated triggers in the presence and absence of 5 μM NSA. The number of NET‐forming neutrophils was determined by counting the DNA‐releasing cells in ten high power fields (*n* = 5). Representative original data (right). Co‐localization of elastase (green) with released DNA (PI, red) is indicated by the yellow color. Bars, 10 μM. (B) Quantification of dsDNA in supernatants of activated neutrophils using PicoGreen fluorescent dye (*n* = 3). (C) Total ROS production by activated human neutrophils in the presence and absence of 5 μM NSA was measured by flow cytometry. During cell activation, cells were incubated with 1 μM DHR123 (*n* = 3). (D) Bacterial killing by colony forming unit (cfu) assay. Human neutrophils exert antibacterial activity against *E. coli* that can be partially blocked by 100 U/mL DNase I both in the presence and absence of 5 μM NSA (*n* = 3). (E) Viability of Jurkat cells was analyzed by ethidium bromide exclusion assay. In parallel experiments, 5 μM NSA blocked cell death induced by 20 ng/mL TNF‐α plus 100 nM Smac mimetic AT‐406 (cIAP1/2 selective IAP‐antagonist) in caspase‐8 deficient and 20 μM zVAD‐FMK–treated Jurkat cells (*n* = 3), demonstrating that the inhibitor was pharmacologically active. All data are shown as mean ± SEM of the indicated number of independent experiments. *p < 0.05; **p < 0.01; ***p < 0.001; one‐way ANOVA test.

Since mouse and human neutrophils may differ in some functional responses, we also performed experiments in the human system, using a pharmacological approach. To trigger NET formation, we stimulated human neutrophils in the same way as mouse neutrophils, including *E. coli* (Fig. 2A). Again, no effect on cell viability upon NET formation was observed in human neutrophils upon NET formation (Supporting Information Fig. 2). The association between extracellular DNA fibers and elastase was considered as defining for NET formation (Fig. 2A, right panels). Quantification of dsDNA release was also verified by PicoGreen fluorescent dye (Fig. 2B). Pharmacological inhibition of MLKL by necrosulfonamide (NSA) had no effect on NET formation (Fig. 2A and B), ROS production (Fig. 2C) or extracellular antibacterial killing activity (Fig. 2D). In contrast, NSA completely prevented TNF‐α/IAP‐antagonist, Smac mimetic AT‐406‐mediated death in caspase‐8‐deficient Jurkat cells, indicating that the compound was functionally active and able to block necroptosis (Fig. 2E).

Taken together, our data clearly show that NET formation by mouse and human neutrophils can occur independently of both RIPK3 and MLKL signaling, and hence independently of necroptosis 6, 7, 8, 9. In fact, this study confirms previous work demonstrating that NET formation does not require cell death to occur at all 16. The idea of a “suicidal NETosis” 17 and the general concept of cell death as a requirement for NET formation both need to be reconsidered.

## Materials and methods

### Antibodies and reagents

Human GM‐CSF was purchased from Novartis Pharma GmbH (Nürnberg, Germany). German glass coverslips (#1 thickness, 12 mm diameter) were purchased from Karl Hecht GmbH & Co. KG “Assistent”, Sondheim/Rhön, Germany. Black, glass‐bottom 96‐well and white 96‐well plates were purchased from Greiner Bio‐One GmbH (Frickenhausen, Germany). Human and mouse C5a were purchased from Hycult Biotech (Uden, The Netherlands). Lipopolysaccharide (LPS, 055:B5), dihydrorhodamine‐123 (DHR123), Saponin, LB medium and LB Agar were from Sigma‐Aldrich (Buchs, Switzerland). Necrosulfonamide (NSA), Phorbol 12‐myristate 13‐acetate (http://en.wikipedia.org/wiki/Phorbol_12‐myristate_13‐acetate PMA), and mouse anti‐GAPDH monoclonal antibody (clone 6C5) were from Merck KGaA (distributed by Millipore, Schaffhausen, Switzerland). DNase I was purchased from Worthington Biochemical Corporation (Lakewood, NJ, USA). Goat anti‐mouse neutrophil elastase polyclonal antibody (M‐18) was from Santa Cruz Biotechnology (distributed by LabForce AG, Nunningen, Switzerland) and mouse anti‐human neutrophil elastase monoclonal antibody (clone NP57) was from Dako (Baar, Switzerland). Quant‐iT^TM^PicoGreen**^®^**dsDNA Assay Kit, propidium iodide (PI), Hanks balanced salt solution (HBSS), RPMI‐1640/GlutaMAX medium, penicillin/streptomycin as well as Alexa Fluor® 488‐conjugated anti‐mouse and anti‐goat secondary antibodies were obtained from ThermoFisher scientific (distributed by LuBioScience GmbH, Lucerne, Switzerland). Human TNF‐α and mouse GM‐CSF were purchased from R&D Systems (Abingdon, UK) and z‐VAD‐FMK (zVAD) from BD Biosciences (Erembodegem, Belgium). Q‐VD was ordered from SM Biochemicals LLC (Anaheim, CA, USA), and mouse TNF‐α was purchased from Enzo Life Sciences (ELS) AG (Lausen, Switzerland). X‐VIVO™ 15 medium with no phenol red and no antibiotics was from Lonza (Verviers, Belgium). Rabbit anti‐mouse RIPK3 polyclonal antibody was from ProSci Inc (Poway, CA, USA). The monovalent Smac mimetic compound AT‐406 was from Selleckchem (Houston, TX, USA) and the ROS‐Glo^TM^H_2_O_2_ kit was purchased from Promega AG (Dübendorf, Switzerland). The protease inhibitor cocktail was from Roche Diagnostics (Rotkreuz, Switzerland). GFP‐labeled *E.coli* M91655 (GFP‐*E. coli*) was a kind gift of Dr. E. Slack (ETH Zurich, Switzerland).

### Neutrophils

Written, informed consent was obtained from all blood donors. The Ethics Committee of the Canton of Bern approved this study. Human blood neutrophils were purified from healthy individuals by Ficoll‐Hypaque centrifugation as previously described 16. Briefly, peripheral blood mononuclear cells (PBMC) were separated by centrifugation on Pancoll Human from PAN^TM^ BioTech (PAN‐Biotech GmbH, Aidenbach, Germany). The lower layer consisting mainly of granulocytes and erythrocytes was treated with erythrocyte lysis solution (155 mM NH_4_Cl, 10 mM KHCO_3_ and 0.1 mM EDTA, pH 7.3). The resulting cell populations contained >95% neutrophils as assessed by staining with the Hematocolor Set (Merck KGaA) followed by light microscopy analysis. Primary bone marrow neutrophils were isolated from wild‐type and *Ripk3*‐deficient mice (C57BL/6N) 18, under animal license 119/2012, by a negative selection technique using an EasySep Mouse Neutrophil Enrichment Kit (Stemcell Technologies, Grenoble, France). Briefly, bone marrow cells were collected by flushing the femur with 5 mL of isolation medium (PBS plus 2% FCS, no EDTA added) using a 26‐gauge needle and filtering through a sterile 70‐μm nylon cell strainer. Bone marrow single‐cell suspensions were then washed with medium and the cells counted with a haemocytometer using the Türk's staining solution (Dr. Grogg Chemie AG, Stettlen, Switzerland). Primary bone marrow neutrophils were then isolated by a negative selection technique using the EasySep Mouse Neutrophil Enrichment Kit (Stemcell Technologies, Grenoble, France) and the isolation medium described above. Neutrophil purity was higher than 93% as assessed by staining with the Hematocolor Set (Merck KGaA) followed by light microscopic analysis 19.

### Immunoblotting

RIPK3 protein expression was analyzed by immunoblotting. 1–5 × 10^6^ cells were washed with PBS and lysed in modified RIPA buffer (50 mM Tris‐HCl pH 8.0, 150 mM NaCl, 1% Triton‐X100, 0.5% Na‐deoxycholate, 0.1% SDS, 2 mM EDTA supplemented with 0.5 mg/mL pepstatin A and complete protease inhibitor cocktail). Protein concentrations were determined by BCA protein assay (Thermo Fisher Scientific) and samples denatured in reducing Laemmle buffer prior to separation by SDS/PAGE gel electrophoresis. Proteins were blotted on PVDF transfer membrane (Merck KGaA) and subsequently probed with the following primary antibodies: Rabbit anti‐mouse RIPK3 polyclonal antibody (1:1000) or mouse anti‐GAPDH monoclonal antibody (1:10 000). Secondary antibodies coupled to horseradish peroxidase were from GE Healthcare Life Sciences (distributed by VWR International GmbH, Dietikon, Switzerland) and signals detected by enhanced chemiluminescence (ECL Western blotting substrate, Thermo Fisher Scientific) and detected on photosensitive film (ECL Hyperfilm, GE Healthcare Life Sciences).

### Confocal laser scanning microscopy and quantification of NET formation

Human neutrophils were resuspended in X‐VIVO™ 15 medium (2.5 × 10^6^/mL) and were pre‐incubated for 30 min in presence or absence of 5 and 15 μM NSA. One hundred microliters of cell suspension was primed with 25 ng/mL GM‐CSF for 20 min on untreated glass coverslips which had previously been washed with acetone, ethanol, ddH_2_O and baked in an oven. Cells were subsequently stimulated with 10^−8^ M C5a or 0.3 μg/mL LPS. Unprimed neutrophils were incubated with *E. coli* (1:3 ratio) or 25 nM PMA for 15 min. Cells were then fixed with 4% paraformaldehyde for 5 min, subsequently washed three times in PBS (pH 7.4), and mounted in ProLong Gold mounting medium. For extracellular DNA detection, cells were stained with 5 μM MitoSOX Red and 1 μg/mL Hoechst 33342.

Neutrophil elastase co‐localization with extracellular DNA was analyzed by indirect immunofluorescence as previously described 20. Briefly, cells were fixed with 4% paraformaldehyde and permeabilized with 0.05% saponin. Monoclonal mouse antibody to human neutrophil elastase (1:1000) or polyclonal goat anti‐mouse neutrophil elastase (1:100) served as primary antibodies. Alexa Fluor® 488 conjugated secondary antibody was used at 1:400. In these experiments, cells were stained with 10 μg/mL PI for extracellular DNA detection.

Slides were examined and images acquired by LSM 700 (Carl Zeiss Micro Imaging, Jena, Germany) using 63×/1.40 Oil DIC objective and followed by analysis with IMARIS software as previously reported 16, 20.

### Quantification of released dsDNA in culture supernatants

Released dsDNA was quantified as previously described 15. Briefly, 2 × 10^6^ neutrophils in 500 μL of X‐VIVO™ 15 medium were stimulated as described above. At the end of the incubation time, a low concentration of DNase I (2.5 U/mL; Worthington) was added for an additional 10 min. Reactions were stopped by addition of 2.5 mM EDTA, pH 8.0. Cells were centrifuged at 200 × *g* for 5 min. One hundred microliters supernatant were transferred to black, glass‐bottom 96‐well plates (Greiner Bio‐One GmbH) and the fluorescent activity of PicoGreen dye bound to dsDNA was excited at 502 nm and the fluorescence emission intensity was measured at 523 nm using a spectrofluorimeter (SpectraMax M2, Molecular Devices, Biberach an der Riß, Germany), according to the instructions described in the Quant‐iT^TM^PicoGreen**^®^** Assay Kit.

### Reactive oxygen species measurements

ROS measurements were performed in stimulated mouse and human neutrophils by two different methods. The fluorescent detection of ROS activity was done by flow cytometry as previously described 16, 20. Briefly, 2 × 10^6^/mL neutrophils were resuspended in X‐VIVO™ 15 medium and 100 μL of cell suspension was preincubated in the presence and absence of different inhibitors for 30 min, before priming with 25 ng/mL GM‐CSF for 20 min. Cells were then stimulated with 10^−8^ M C5a or 100 ng/mL LPS. As a control, we also stimulated neutrophils with 25 nM PMA for 15 min in the absence of GM‐CSF priming. Dihydrorhodamine 123 (DHR 123) was added to the cells at the final concentration of 1 μM. The reaction was stopped by adding 200 μL of ice‐cold PBS, and ROS activity of the samples was measured immediately by flow cytometer (BD FACSCalibur) and quantified using FlowJo software (Ashland, OR, USA).

ROS was also quantified using the ROS‐Glo^TM^H_2_O_2_ kit (Promega AG), a luminescent assay that can measure H_2_O_2_ levels directly in cell cultures. Briefly, 100 μL of 2.5 × 10^6^/mL neutrophils in X‐VIVO™ 15 medium were transferred to white 96‐well plates (Greiner Bio‐One GmbH) and 25 μM of H_2_O_2_ substrate was added to the cell suspension. Cells were activated as described above. ROS‐Glo detection solution (D‐cysteine, Signal Enhancer solution and Luciferin detection solution, provided by the kit) was then added and incubated for 20 min at room temperature, protected from light. Luminescence activity was detected by GloMax® Explorer machine (Promega AG, Dübendorf, Switzerland).

### Bacterial killing assay

The bacterial killing assay was performed as previously described 19. Briefly, a single colony of GFP‐*E. coli* was cultured in Luria broth base (LB) medium at 37°C, shaking at 220 r.p.m. overnight. On the next day, the bacterial culture was diluted 1:100 in LB medium and grown until a mid‐logarithmic growth phase (OD^600^ = 0.7). Following centrifugation at 1000 × *g* for 5 min, bacterial pellets were washed twice with 1× Hank's Balanced Salt Solution (HBSS; LuBioScience GmbH) and centrifuged at 100 × *g* for 5 min to remove any clumped bacteria. Bacteria were opsonized with 10% mouse sera (heat‐inactivated) in 1 × HBSS, rotating end‐over‐end (6 r.p.m.) for 20 min at 37°C and used immediately. Activated neutrophils (1 × 10^7^/mL in RPMI 1640 plus 2% FCS) were mixed with an equal volume of opsonized bacteria (5 × 10^7^/mL) in the presence and absence of 100 U/mL DNase I. The co‐cultures of cells and bacteria were rotated end‐over‐end (6 r.p.m.) for 30 min at 37°C. At the end of the incubation period, an equal volume of ice‐cold 1 × HBSS was added to each tube to stop the reaction, and cells were pelleted by gentle centrifugation (5 min, 100 × *g*, 4°C) using a swing‐out rotor. Supernatants containing bacteria were collected, diluted 1:300, plated on agar and grown overnight before counting the colonies. The tubes containing bacteria alone were treated the same way and used as controls.

### Induction of necroptosis and viability measurements

Necroptosis was induced in 0.5 × 10^6^ cells/mL mouse neutrophils cultured in complete culture medium (RPMI 1640 plus GlutaMAX supplemented with 5% FCS) by adding 1 μM Smac mimetic AT‐406 and 20 μM Q‐VD for 30 min, before stimulating with 100 ng/mL mouse TNF‐α. Cell viability was determined after 24 and 48 h by ethidium bromide (25 μM) exclusion as assessed by flow cytometry (BD FACSCalibur).

Functional inhibition of MLKL was controlled by measuring viability in TNF‐α treated caspase‐8‐deficient Jurkat cells (kind gift of Dr. T. Brunner, University of Konstanz, Germany). Briefly, caspase‐8‐deficient Jurkat cells (1 × 10^6^/mL) were cultured in complete culture medium with 20 ng/mL recombinant human TNF‐α, in the presence or absence of 100 nM Smac mimetic AT‐406, 20 μM z‐VAD, and 5 μM NSA. Cell viability was determined after 24 and 48 h by an ethidium bromide exclusion test as described above.

### Statistical analysis

Mean values (± SEM) are provided. To compare groups, one‐way ANOVA was applied. *P* values < 0.05 were considered statistically significant.

## Conflict of interest

The authors declare no financial or commercial conflict of interest.

## Supporting information

As a service to our authors and readers, this journal provides supporting information supplied by the authors. Such materials are peer reviewed and may be re‐organized for online delivery, but are not copy‐edited or typeset. Technical support issues arising from supporting information (other than missing files) should be addressed to the authors.

Supplementary Material

Peer Review Correspondence

## References

[1] Simon, D. , Simon, H. U. and Yousefi, S. , Extracellular DNA traps in allergic, infectious, and autoimmune diseases. Allergy 2013 68: 409–416. 23409745 10.1111/all.12111

[2] Brinkmann, V. , Reichard, U. , Goosmann, C. , Fauler, B. , Uhlemann, Y. , Weiss, D. S. , Weinrauch, Y. **et al**, Neutrophil extracellular traps kill bacteria. Science 2004 303: 1532–1535. 15001782 10.1126/science.1092385

[3] Urban, C. F. , Reichard, U. , Brinkmann, V. and Zychlinsky, A. , Neutrophil extracellular traps capture and kill *Candida albicans* yeast and hyphal forms. Cell. Microbiol. 2006 8: 668–676. 16548892 10.1111/j.1462-5822.2005.00659.x

[4] Lande, R. , Ganguly, D. , Facchinetti, V. , Frasca, L. , Conrad, C. , Gregorio, J. , Meller, S. **et al**, Neutrophils activate plasmacytoid dendritic cells by releasing self‐DNA‐peptide complexes in systemic lupus erythematosus. Sci. Transl. Med. 2011 3: 73ra19. 10.1126/scitranslmed.3001180 PMC3399524 21389263

[5] Garcia‐Romo, G. S. , Caielli, S. , Vega, B. , Connolly, J. , Allantaz, F. , Xu, Z. , Punaro, M. **et al**, Netting neutrophils are major inducers of type I IFN production in pediatric systemic lupus erythematosus. Sci. Transl. Med. 2011 3: 73ra20. 10.1126/scitranslmed.3001201 PMC3143837 21389264

[6] Pasparakis, M. and Vandenabeele, P. , Necroptosis and its role in inflammation. Nature 2015 517: 311–320. 25592536 10.1038/nature14191

[7] He, S. , Wang, L. , Miao, L. , Wang, T. , Du, F. , Zhao, L. and Wang, X. Receptor interacting protein kinase‐3 determines cellular necrotic response to TNF‐alpha. Cell 2009 137: 1100–1111. 19524512 10.1016/j.cell.2009.05.021

[8] Zhang, D. W. , Shao, J. , Lin, J. , Zhang, N. , Lu, B. J. , Lin, S. C. , Dong, M. Q. **et al**, RIP3, an energy metabolism regulator that switches TNF‐induced cell death from apoptosis to necrosis. Science 2009 325: 332–336. 19498109 10.1126/science.1172308

[9] Cho, Y. S. , Challa, S. , Moquin, D. , Genga, R. , Ray, T. D. , Guildford, M. and Chan, F. K. Phosphorylation‐driven assembly of the RIP1‐RIP3 complex regulates programmed necrosis and virus‐induced inflammation. Cell 2009 137: 1112–1123. 19524513 10.1016/j.cell.2009.05.037 PMC2727676

[10] Wong, E. W. L. , Vince, J. E. , Lalaoui, N. , Lawlor, K. E. , Chau, D. , Bankovacki, A. , Anderton, H. **et al**, cIAPs and XIAP regulate myelopoiesis through cytokine production in an RIPK1‐ and RIPK3‐dependent manner. Blood 2014 123: 2562–2572. 24497535 10.1182/blood-2013-06-510743

[11] Lawlor, K. E. , Khan, N. , Mildenhall, A. , Gerlic, M. , Croker, B. A. , D'Cruz, A. A. , Hall, C. **et al**, RIPK3 promotes cell death and NLRP3 inflammasome activation in the absence of MLKL. Nat. Commun. 2015 6: 6282. 25693118 10.1038/ncomms7282 PMC4346630

[12] Fuchs, T. A. , Abed, U. , Goosmann, C. , Hurwitz, R. , Schulze, I. , Wahn, V. , Weinrauch, Y. **et al**, Novel cell death program leads to neutrophil extracellular traps. J. Cell Biol. 2007 176: 231–241. 17210947 10.1083/jcb.200606027 PMC2063942

[13] Brinkmann, V. and Zychlinsky, A. , Beneficial suicide: why neutrophils die to make NETs. Nat. Rev. Microbiol. 2007 5: 577–582. 17632569 10.1038/nrmicro1710

[14] Remijsen, Q. , Kuijpers, T. W. , Wirawan, E. , Lippens, S. , Vandenabeele, P. and Van den Berghe, T. , Dying for a cause: NETosis, mechanisms behind an antimicrobial cell death modality. Cell Death Differ. 2011 18: 581–588. 21293492 10.1038/cdd.2011.1 PMC3131909

[15] Neumann, A. , Brogden, G. , Jerjomiceva, N. , Brodesser, S. , Naim, H. Y. and von Köckritz‐Blickwede, M. , Lipid alterations in human blood‐derived neutrophils lead to formation of neutrophil extracellular traps. Eur. J. Cell Biol. 2014 93: 347–354. 25172775 10.1016/j.ejcb.2014.07.005

[16] Yousefi, S. , Mihalache, C. , Kozlowski, E. , Schmid, I. and Simon H. U. , Viable neutrophils release mitochondrial DNA to form neutrophil extracellular traps. Cell Death Differ. 2009 16: 1438–1444. 19609275 10.1038/cdd.2009.96

[17] Yipp, B. G. and Kubes, P. , NETosis: how vital is it? Blood 2013 122: 2784–2794. 24009232 10.1182/blood-2013-04-457671

[18] Newton, K. , Sun, X. and Dixit, V. M. , Kinase RIP3 is dispensable for normal NF‐kappa Bs, signaling by the B‐cell and T‐cell receptors, tumor necrosis factor receptor 1, and Toll‐like receptors 2 and 4. Mol. Cell Biol. 2004 24: 1464–1469. 14749364 10.1128/MCB.24.4.1464-1469.2004 PMC344190

[19] Rozman, S. , Yousefi, S. , Oberson, K. , Kaufmann, T. , Benarafa, C. and Simon, H. U. , The generation of neutrophils in the bone marrow is controlled by autophagy. Cell Death Differ. 2015 22: 445–456. 25323583 10.1038/cdd.2014.169 PMC4326574

[20] Morshed, M. , Hlushchuk, R. , Simon, D. , Walls, A. F. , Obata‐Ninomiya, K. , Karasuyama, H. , Djonov, V. **et al**, NADPH oxidase‐independent formation of extracellular DNA traps by basophils. J. Immunol. 2014 192: 5314–5323. 24771850 10.4049/jimmunol.1303418

