# Supplementary material for: NET formation can occur independently of RIPK3 and MLKL signaling
Source: Eur J Immunol. 2015 Dec 2;46(1):178–84. doi: 10.1002/eji.201545615 (PMC4738457; doi:10.1002/eji.201545615)
Supplement: Supplementary file 1 — Supplementary Material [file EJI-46-178-s001.pdf]

European Journal of Immunology

Supporting Information

for

DOI 10.1002/eji.201545615

Poorya Amini, Darko Stojkov, Xiaoliang Wang, Simone Wicki, Thomas Kaufmann, Wendy Wei-Lynn Wong, Hans-Uwe Simon and Shida Yousefi

NET formation can occur independently of RIPK3 and MLKL signaling

© 2015 The Authors. *European Journal of Immunology* published by WILEY-VCH Verlag GmbH & Co. KGaA, Weinheim

Supporting Information

NET formation can occur independently of RIPK3 and MLKL signaling

Poorya Amini[1], Darko Stojkov[1], Xiaoliang Wang[1], Simone Wicki[1], Thomas Kaufmann[1], Wendy Wei-Lynn Wong[2], Hans-Uwe Simon[1], and Shida Yousefi[1]

[1]Institute of Pharmacology, University of Bern, Bern, Switzerland
[2]Institute of Experimental Immunology, University of Zurich, Zurich, Switzerland

Supporting Information Figure 1

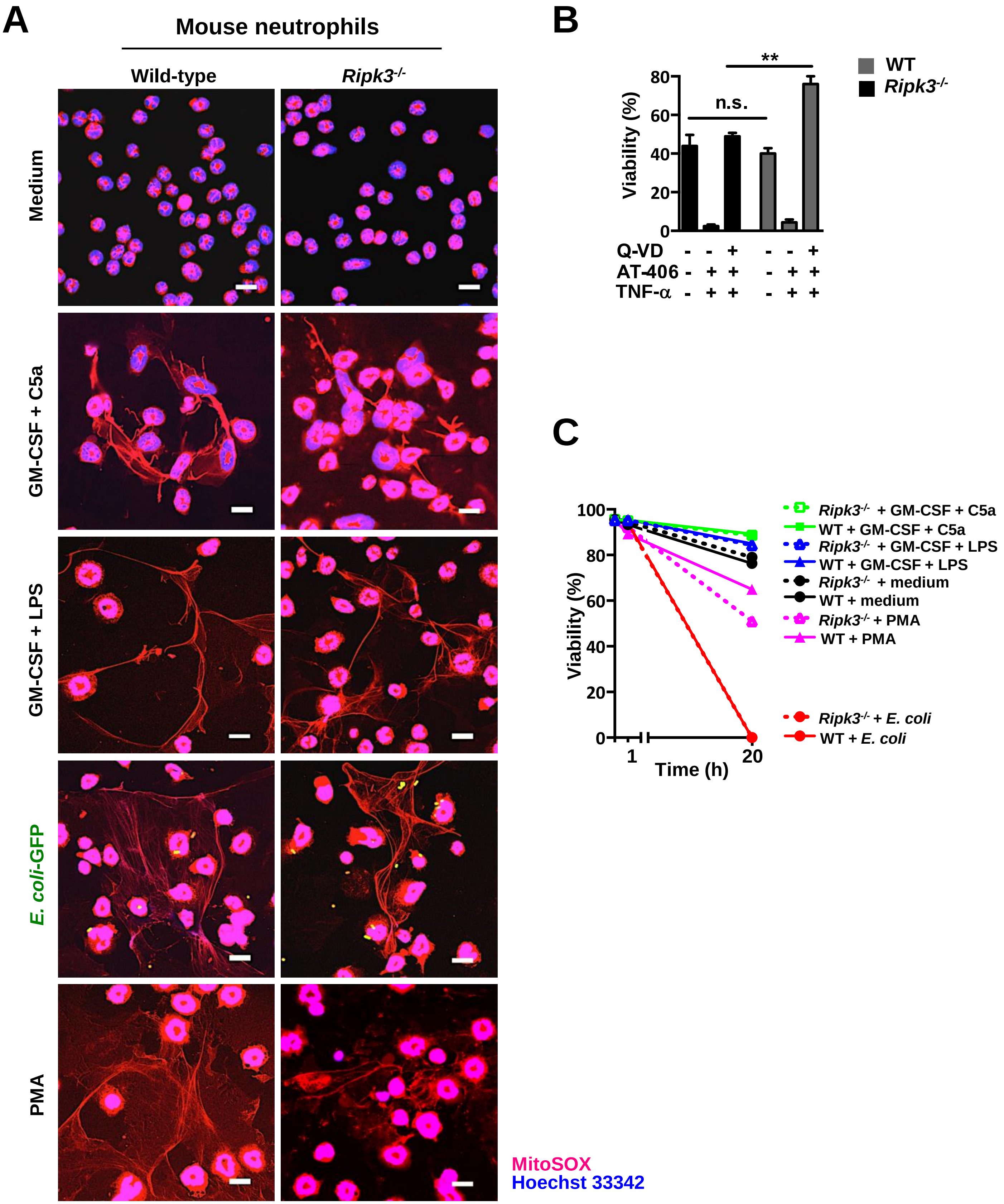

Supporting Information Figure 1. Activated mouse *RIPK3*-deficient neutrophils can release DNA in the absence of cell death.

Confocal microscopy. NET formation following short-term stimulation (total 45 min) of mouse neutrophils with the indicated triggers. No difference was observed between wild-type and *Ripk3*-deficient cells. Representative images of three independent experiments are shown. Bars, 10 μm. The number of DNA-releasing neutrophils was determined in ten high power fields (Fig. 1B and Supporting Information Movie 1). (B) Flow cytometry. Mouse neutrophils were incubated in presence or absence of 1 μM Smac mimetic AT-406 and 20 μM Q-VD for 30 min, before 100 ng/ml mouse TNF-α stimulation. Cell viability is shown after 48 h (n=3). The difference between AT-406 and Q-VD pre-treated wild-type and *Ripk3*-deficient neutrophils following TNF-α stimulation represents the percentage necroptotic cells seen in wild-type neutrophils only. **, $p<0.01$. (C) Flow cytometry. Wild-type and *Ripk3*-deficient neutrophils were stimulated as indicated. Cell viability was measured after 1 and 20 h (n=3). No significant cell death is induced within 1 h, independent of the NET-forming trigger including 25 nM PMA.

Supporting Information Movie 1

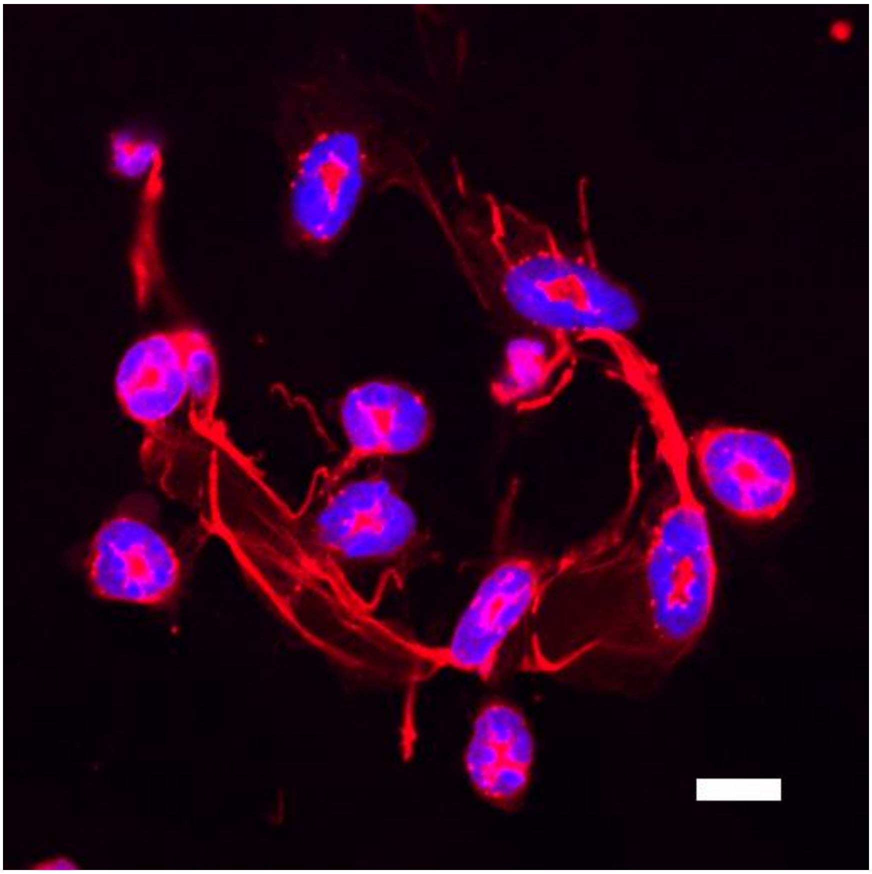

Mouse neutrophils: GM-CSF + C5a

GM-CSF-primed and C5a-activated mouse neutrophils release DNA in the absence of cell death.
Mouse neutrophils seeded on glass coverslips were incubated with GM-CSF for 20 min and subsequently activated with C5a for 15 min. In the last 5 min of the stimulation period, cells were labeled with 5 mM MitoSOX Red to stain the extracellular DNA. Cells were then fixed with 4% paraformaldehyde and the nucleus stained with 1 μM Hoechst 33342. The image was acquired by LSM 700 (Carl Zeiss Micro Imaging, Jena, Germany) using an 63x /1.40 Oil DIC objective with an interval of 0.1 µm, slices taken throughout the z-axis (z stacks) with 27 slices per stack in order to show extracellular DNA in its full extension. Bar, 10 μm.

Supporting Information Figure 2

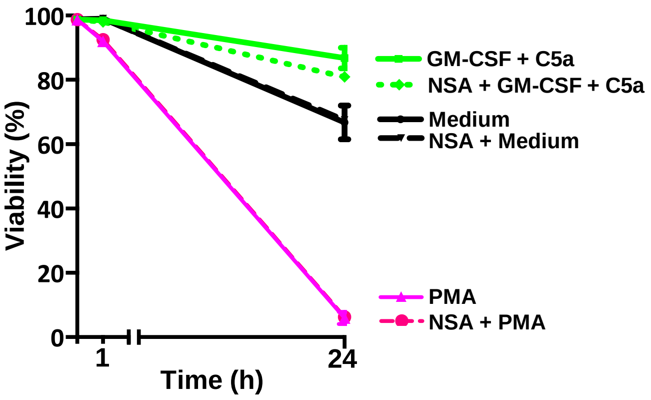

Supporting Information Figure 2. Human neutrophils do not exhibit cell death in association with DNA trap formation.

Flow cytometry. Control and NSA-treated human neutrophils were stimulated as indicated. Cell viability was measured after 1 and 24 h (n=3). No significant cell death is induced within 1 h, independent of the NET-forming trigger including 25 nM PMA.
