# Supplementary material for: NET formation can occur independently of RIPK3 and MLKL signaling
Source: Eur J Immunol. 2015 Dec 2;46(1):178–84. doi: 10.1002/eji.201545615 (PMC4738457; doi:10.1002/eji.201545615)
Supplement: Supplementary file 2 — Peer Review Correspondence [file EJI-46-178-s002.pdf]

European Journal of Immunology

Peer review correspondence

NET formation can occur independently of RIPK3 and MLKL signaling

Poorya Amini, Darko Stojkov, Xiaoliang Wang, Simone Wicki, Thomas Kaufmann, Wendy Wei-Lynn Wong, Hans-Uwe Simon and Shida Yousefi

Corresponding author: Shida Yousefi, Institute of Pharmacology, University of Bern, Bern, Switzerland

---

Review Timeline:	Submission date:	2 March 2015
	First editorial decision:	30 March 2015
	First revision received:	23 June 2015
	Second editorial decision:	17 July 2015
	Authors' appeal:	20 July 2015
	Third editorial decision:	14 September 2015
	Second revision received:	5 October 2015
	Accepted:	3 November 2015

Handling Executive Committee members: Prof. Iain McInnes

Please note that the correspondence below does not include the standard editorial instructions regarding preparation and submission of revised manuscripts, only the scientific revisions requested and addressed.

First Editorial Decision – 30 March 2015

Dear Prof. Simon,

Manuscript ID eji.201545615 entitled "The formation of neutrophil extracellular traps does not depend on the RIPK3-MLKL signaling pathway" which you submitted to the European Journal of Immunology has been reviewed.

The comments of the referees are included at the bottom of this letter. Even though referee #1 has suggested rejection and that the other referees have some concerns, the Executive Editor would like to see a revised version of your study. The edited manuscript, taking into account the comments of all the referees, will be reconsidered for publication.

You should also pay close attention to the editorial comments included below. **In particular, please edit your figure legends to follow Journal standards as outlined in the editorial comments. Failure to do this will result in delays in the re-review process.**

Please note that submitting a revision of your manuscript does not guarantee eventual acceptance, and that your revision will be re-reviewed by the referee(s) before a decision is rendered.

If the revision of the paper is expected to take more than three months, please inform the editorial office. Revisions taking longer than six months may be assessed by new referee(s) to ensure the relevance and timeliness of the data.

Once again, thank you for submitting your manuscript to European Journal of Immunology and we look forward to receiving your revision.

Yours sincerely,
Laura Soto Vazquez

On behalf of Prof. Iain McInnes

Editorial Office
European Journal of Immunology
e-mail: ejied@wiley.com
www.eji-journal.eu

****

Reviewer: 1
Comments to the Author
It is of great concern that this work is superficial, and how articificial the experimental systems are, which include major use of a transformed neutrophil cell line, and GM-CSF priming of blood neutrophils, which both do not appear necessary and could well have tainted the results, which are unconvincing to this reader. The narrow scope of this work, including lack of in vivo studies, further reflects the lack of definitive depth of the experimental approach.

Reviewer: 2
Comments to the Author

In their manuscript "The formation of neutrophil extracellular traps does not depend on the RIPK3-MLKL signaling pathway" Amini and colleagues investigated the role of the necroptosis pathway in the formation of extracellular traps. In face of the importance of NETosis the topic is of extremely high importance, if the analyses are performed in a proper way.

Criticism:

The manuscript is rather incomplete.

The general statement of the title is a overinterpretation of a very limited amount of in vitro experiments not even employing primary cells in all cases.

The Material and method section does not carefully describe the exact procedure (medium, supplement, precoating culture conditions and so on).

Instead of Hoxb8 neutrophil precursor cells in vitro differentiated into "mature neutrophils" primary neutrophils have to be used for the study.

Figure 1A Blot is lousy

Figure 1B is not convincing. The authors should provide better material, at least for the reviewing process.

Figure 2B the cell densities of the cultures displayed in Fig 2B are different excluding proper quantification.

To obtain reliable data quantification of NETosis has to be performed with several independent techniques (some of these techniques should employ at least 1000 cells per sample in quintaplicates to carefully quantify NETosis).

The list of references is biased and ignors many important papers.

Reviewer: 3

Comments to the Author

Whether NETosis involves necroptosis is an interesting topic that should be investigated. However the authors here, use this work to support their preferred controversial hypothesis stemming from their work in eosinophils to try to provide evidence that NETs are not released by dying cells. Necroptosis is only one of several forms of death and many novel mechanisms are being constantly identified. Death has been demonstrated beyond doubt and it does not necessarily have to occur via the necroptosis pathway. Apoptosis does not require RIPK3. If the authors rational were to be correct, apoptosis does not involve cell death!

The authors perform the right experiments but their methods need to be much improved and there are serious issues and inconsistencies regarding the quality of the data presented. Therefore, it is not possible to draw solid conclusions with the data provided.

Major issues:

European Journal of Immunology

1) In the introduction the authors state that:

“While most investigators agree that NETs contribute significantly to innate immunity, the molecular mechanisms responsible for their formation remain unclear and in dispute. The focus is a simple question: Does the neutrophil need to die in order to provide the extracellular DNA scaffold characteristic for NETs?”

There is a large body of literature on the molecular mechanism of NETosis by the Zychlinsky lab alongside work by Paul Kubes which have examined whether NETosis involves a cell death processes. According to this work, during sepsis in vivo, the majority of neutrophils undergo death, although there might be a small fraction of cells first arriving to the site that catapult their nuclei and somehow mysteriously do not burst. However the molecular mechanism that triggers membrane permeabilization and death is not clear, ie whether it depends on the necroptosis pathways. I think it is important to clarify this in the introduction.

2) Fig. 1A It would be great if the authors could provide a better immunoblot.

3) Fig. 1B. I could not find any description for how the authors quantitated NET formation. I suspect they counted NETs on fixed images by eye. It is unclear to me how the authors obtained the quantitative data from images such as the ones shown in the right panel. For example, the authors report 40% NET formation efficiency with PMA. But the stained image shows only 1 out of 34 neutrophils making NETs. All the remaining neutrophils are intact.

4) A more general comment: It is extremely challenging to induce NETosis in mouse neutrophils with PMA, let alone with E. coli that does not even induce NETosis in human blood-derived primary neutrophils. Therefore, I am surprised by the reported efficiencies in NETosis presented here. Fungi that are the most potent microbial NETosis trigger alongside P. aeruginosa will only yield 20% NETing neutrophils at best.

5) Fig. 1C. The DHFR data show very high background and only a 2-fold increase in fluorescence. While, it is clear that there are no differences in WT and Ripk3-/- neutrophils, the assay may not be robust enough to detect them. Luminol might be better.

6) The high background fluorescence might signify reactivation. The ficoll purification protocol is known to preactivate neutrophils and background NETosis is high in the absence of stimulation. It should therefore be avoided as it would skew the results and mask a potential physiological deficiency in NETosis. Percoll is much preferred.

7) The same concerns apply for Fig. 2

European Journal of Immunology

First revision – authors' responses – 23 June 2015

Response to Reviewer 1

It is of great concern that this work is superficial, and how articificial the experimental systems are, which include major use of a transformed neutrophil cell line, and GM-CSF priming of blood neutrophils, which both do not appear necessary and could well have tainted the results, which are unconvincing to this reader. The narrow scope of this work, including lack of in vivo studies, further reflects the lack of definitive depth of the experimental approach.

Answer: We try to understand the concern that the Reviewer 1 has risen. We are not using a transformed neutrophil cell line but rather ex vivo differentiated bone marrow derived neutrophils (first described by Wang & Kamps, Nature Methods, 2006). These cells are capable of executing normal differentiation and innate immune function. However, to address the Reviewer 1 concern, we have now replaced all data presented in Fig. 1 using primary mature mouse neutrophils, purified from bone marrow of wild-type and Ripk3 ko mice (please see Fig 1A, 1B, 1C, 1D, 1E, 1F, 1G).
GM-CSF priming and subsequent activation of neutrophils have been reported repeatedly to prime neutrophils and cause degranulation under in vivo and in vitro conditions (van Pelt & van Oers, Blood, 1996) & (Fossati & Edwards, Annual Rheumatology and Disease, 2001). In many, if not most, physiological scenarios of infections, neutrophils will sooner or later be primed by proinflammatory cytokines, including GM-CSF. We intentionally selected this type of activation method to mimic the in vivo condition for NET formation. We have also included PMA (25 nM) activation for comparison.
We hope that current data presented in this manuscript is of significant value for better understanding of mechanism of NET formation.

Response to Reviewer 2

In their manuscript "The formation of neutrophil extracellular traps does not depend on the RIPK3-MLKL signaling pathway" Amini and colleagues investigated the role of the necroptosis pathway in the formation of extracellular traps. In face of the importance of NETosis the topic is of extremely high importance, if the analyses are performed in a proper way.

Answer: We greatly appreciate the comments of Reviewer 2 and we would like to thank for his/her suggestions for further improvement of our manuscript.

The manuscript is rather incomplete. The general statement of the title is an overinterpretation of a very limited amount of in vitro experiments not even employing primary cells in all cases.

European Journal of Immunology

Answer: We have now included experimental data using primary mature mouse neutrophils isolated from bone marrow of wild-type and RIPK3 ko mice (please see Fig 1A, 1B, 1C, 1D, 1E, 1F, 1G).

The Material and method section does not carefully describe the exact procedure (medium, supplement, precoating culture conditions and so on).

Answer: We have now provided more detailed experimental protocols.

Instead of Hoxb8 neutrophil precursor cells in vitro differentiated into “mature neutrophils” primary neutrophils have to be used for the study.

Answer: We have now included experimental data using primary mature mouse neutrophils isolated from bone marrow of wild-type and Ripk3 ko mice (please see Fig 1A, 1B, 1C, 1D, 1E, 1F, 1G).

Figure 1A Blot is lousy

Answer: We have now provided a better quality of immunoblot demonstrating the lack of RIPK3 protein expression in mouse bone marrow cells isolated from Ripk3 ko mouse (please see new Fig. 1A).

Figure 1B is not convincing. The authors should provide better material, at least for the reviewing process.

Answer: As suggested by the Reviewer 2, we replaced Figure 1B (please see new Fig. 1B, and Fig. 1C), using primary mature mouse neutrophils isolated from bone marrow. For quantification of released DNA, we routinely stain the cells with MitoSOX red and Hoechst. Using this type of staining method, we have the highest detection sensitivity for released DNA, and at the same time we can observe the morphology of the nuclei. As shown in our data, activated neutrophils all retain intact nuclei. Please note that cells release extracellular DNA in a 3-dimensional fashion (above the microscopy focus level as well). We quantify neutrophils with released DNA by changing the focal layers, allowing us to observe all the participating neutrophils in the process of NET formation.

The purpose of figure 1B was to demonstrate that NETs consist of DNA and granule proteins, namely neutrophil elastase. Therefore, the samples depicted in Figure 1C & 2A (right side), were stained differently, using propidium iodide. PI dye is less sensitive for detection of released DNA than MitoSOX (strongly detects oxidized DNA, above pictures). Please note that in the revised manuscript, we now included an additional method to quantify released DNA in cell culture supernatants using PicoGreen, a dsDNA binding fluorescent dye (Fig. 1D and Fig. 2B). However, it is important to note that simply assaying

European Journal of Immunology

cell-free DNA should not be accepted as sufficient evidence for NET formation, and microscopic quantification remains indispensible.

Figure 2B the cell densities of the cultures displayed in Fig 2B are different excluding proper quantification. To obtain reliable data quantification of NETosis has to be performed with several independent techniques (some of these techniques should employ at least 1000 cells per sample in quintaplicates to carefully quantify NETosis).

Answer: We believe the reviewer meant Figure 2A, as figure 2B is quantification of ROS activity. Figure 2A has been quantified (right hand side), however, to address the Reviewer's concern, we now added a new method to quantify released DNA in cell culture supernatants using PicoGreen, a dsDNA-binding fluorescent dye (please see new Fig. 2B; and also new Fig. 1D). In addition we have improved the microscopy images in Fig. 1C and 2 A, right hand side.

The list of references is biased and ignores many important papers.

Answer: We are limited to 10 references in the Letters to the editor format. However, to satisfy Reviewer 2, we removed some of our own published references and included other recent publications in this field.

Response to Reviewer 3

Whether NETosis involves necroptosis is an interesting topic that should be investigated.
However the authors here, use this work to support their preferred controversial hypothesis stemming from their work in eosinophils to try to provide evidence that NETs are not released by dying cells. Necroptosis is only one of several forms of death and many novel mechanisms are being constantly identified. Death has been demonstrated beyond doubt and it does not necessarily have to occur via the necroptosis pathway. Apoptosis does not require RIPK3. If the authors rational were to be correct, apoptosis does not involve cell death!

Answer: The exact molecular mechanism of NET formation is still not well established and under intense investigation. We are not challenging the idea that dying cells release nuclear DNA. We have proposed that live cells are also capable of releasing DNA while staying alive for prolonged times thereafter. We agree that under certain pathological conditions neutrophils release nuclear DNA. For example, neutrophils are reported to release nuclear DNA upon encountering bacteria capable of secreting pore-forming enzymes / toxins. This type of nuclear DNA release could occur as early as 5 min (Malachowa & DeLeo, J. Immunol., 2013) & (Pilsczek & Kubes, J. Immunol., 2010) & (Aulik & Czuprynski, Infection & Immunity, 2010). However, under physiological condition in healthy individuals, nuclear DNA release upon

European Journal of Immunology

neutrophil activation or microorganisms encounter is still controversial and the question has been raised whether NETs (if it consists of nuclear DNA) were at all beneficial to the host (Yipp & Kubes, Blood, 2013) & (Nauseef, J. Leukocyte Biol., 2012). NETosis has been reported to participate in pathogenesis of autoimmune and inflammatory disorders, with proposed involvement in glomerulonephritis, chronic lung disease, sepsis, and vascular disorders (Kaplan & Radic, J. Immunol., 2012). Therefore, it is important to explore all potential mechanisms of NET formation.

Others and we have already reported that apoptosis or necrosis are not involved in process of NET formation (Remijsen & Vandenabeele, Cell Research, 2011) & (Yousefi & Simon, Cell Death Differ., 2009). Addition of Q-VD-oph (pan-caspase inhibitor) or Nec-1 (inhibitor of RIP1 kinase and RIP1-dependent necrosis) did not prevent NET formation, and now we report that NSA (inhibitor of RIPK3-MLKL signaling pathway, necroptosis) similarly does not affect NET formation. Furthermore, we routinely test the viability of cells before and after NET formation and observe no increase of cell death in comparison to control, after short (1 h) or longer (24 h) incubation. Our present manuscript would eliminate involvement of one more type of cell death, namely necroptosis in the process of NETs formation.

We agree with the Reviewer that cell death, so-called NETosis, does occur upon exposure of cells to high concentration of PMA (100-200 nM) for 2-4 hours. This type of cell death had been already reported two decades ago (Takei & Sendo, J. Leukocyte Biol., 1996). Our interest is to understand the process of NET formation as it was elegantly reported for the first time (Brinkmann & Zychlinsky, Science, 2004). Looking into details of the experimental protocols in this paper (Science 2004, Suppl. Info.), the legend to Figure S1 states: “at a concentration of 10 nM PMA, DNA is extruded as early as 10 min” and maximum activation time was 30 min. It is clear that “cell death” (i.e. NETosis) could not have occurred in such a short time at such low concentration of PMA. Therefore we strongly believe we should be given the chance to report our observations, challenging the NETosis theory. Furthermore, we hope this type of healthy skepticism encourages scientists in this field to pay more attention to detailed experimental procedures and conclusions.

The authors perform the right experiments but their methods need to be much improved and there are serious issues and inconsistencies regarding the quality of the data presented. Therefore, it is not possible to draw solid conclusions with the data provided.

Answer: We appreciate the reviewer’ encouragements regarding the “right experiments”, and follow his/her advise to improve the methods (please see below).

2) Fig. 1A It would be great if the authors could provide a better immunoblot.

Answer: We have now provided a better immunoblot demonstrating the lack of RIPK3 protein expression in wild-type and Ripk3 ko mouse bone marrow cells (please see new Fig. 1A).

European Journal of Immunology

3) Fig. 1B. I could not find any description for how the authors quantitated NET formation. I suspect they counted NETs on fixed images by eye. It is unclear to me how the authors obtained the quantitative data from images such as the ones shown in the right panel. For example, the authors report 40% NET formation efficiency with PMA. But the stained image shows only 1 out of 34 neutrophils making NETs. All the remaining neutrophils are intact.

Answer: We count cells under the microscope in at least 10 high power fields and by acquiring z-stacks to capture all the cells that have released DNA. Released DNA is contributed by many neutrophils in the field, and the graph is representing NET-forming neutrophils. We count only those neutrophils actively involved in DNA release. We have replaced the microscopy image (new Fig. 1C) using primary mature mouse neutrophils isolated from bone marrow, and additionally we also quantified released DNA in cell culture supernatant using PicoGreen dsDNA binding fluorescent dye (please see new Fig. 1D, and 2B).

4) A more general comment: It is extremely challenging to induce NETosis in mouse neutrophils with PMA, let alone with E. coli that does not even induce NETosis in human blood-derived primary neutrophils. Therefore, I am surprised by the reported efficiencies in NETosis presented here. Fungi that are the most potent microbial NETosis trigger alongside P. aeruginosa will only yield 20% NETing neutrophils at best.

Answer: We completely agree with the Reviewer 3 that E. coli does not induce NETosis, but it causes NET formation. One reason PMA cannot easily kill mouse neutrophils is that mouse neutrophils have lower levels of ROS activity in comparison to human neutrophils. Therefore, they are less able to augment ROS activity to a lethal dose even at relatively high PMA concentration and longer incubation. But augmentation of ROS via physiological activation could still lead to DNA and granule protein release, as expected under physiological conditions.
Bacteria, including E. coli are potent inducer of DNA release in our hand as well as others (Yost & Zimmerman, Blood, 2009) & (Grinberg & Shpigel, Infection and Immunity, 2008).

5) Fig. 1C. The DHFR data show very high background and only a 2-fold increase in fluorescence. While, it is clear that there are no differences in WT and Ripk3-/- neutrophils, the assay may not be robust enough to detect them. Luminol might be better.

Answer: ROS measurement using dihydrorhodamine-123 (DHR123) by flow cytometry is very cost effective and has been well established in our laboratory. Although detection sensitivity of DHR123 fluorescent dye is lower than luminescent assay, it is reliable and consistently shows the differences between activated and un-activated samples. Important point to note is that all physiological stimuli cause moderate increase in ROS activity, in comparison to ROS induced by PMA. However, to satisfy the

Reviewer 3 we have also measured the ROS activity using a luminescent assay (please see new Fig 1F) where we used primary mature mouse neutrophils.

6) The high background fluorescence might signify reactivation. The ficoll purification protocol is known to preactivate neutrophils and background NETosis is high in the absence of stimulation. It should therefore be avoided as it would skew the results and mask a potential physiological deficiency in NETosis. Percoll is much preferred.

Answer: Purified neutrophils after isolation are 99% viable. We have tested Percoll purification method and found no difference in the results. Furthermore, please note that mouse neutrophils (represented in Fig. 1) were not purified by Ficoll. Therefore, our findings could not be due to a wrong neutrophil isolation method.

Second Editorial Decision – 17 July 2015

Dear Prof. Simon,

You recently submitted your revised Manuscript ID eji.201545615.R1 entitled "The formation of neutrophil extracellular traps does not depend on the RIPK3-MLKL signaling pathway" to European Journal of Immunology. Your manuscript has been re-reviewed. Unfortunately, the referees were not satisfied with the revisions made and feel that the manuscript has not improved sufficiently to warrant publication in the Journal (please also see the enclosed comments).

We regret to have to inform you about this decision. However, in view of the great pressure for space at present, only those papers that receive very favorable reviews are eligible for the European Journal of Immunology.

Thank you for considering European Journal of Immunology for the publication of your research. We hope that the outcome of this specific submission will not discourage you from submitting future manuscripts.

Please note that all files associated with this submission (i.e. the manuscripts files that you uploaded to Manuscript Central) but not the manuscript record itself will be deleted in 60 days. We will therefore not be able to enter into any specific queries regarding the manuscript after this date.

Yours sincerely,
Katharina Schmidt

On behalf of Prof. Iain McInnes

Dr. Katharina Schmidt
Editorial Office
European Journal of Immunology
ejied@wiley.com

****

Reviewer: 2
Comments to the Author
In their manuscript "The formation of neutrophil extracellular traps does not depend on the RIPK3-MLKL signaling pathway” Amini and colleagues investigated the role of the necroptosis pathway in the formation of extracellular traps. In face of the importance of NETosis the topic is of extremely high importance, if the analyses are performed in a proper way.
The manuscript is still rather incomplete.
The general statement of the title is still an overinterpretation of a very limited amount of in vitro experiments.
There is no doubt that there are reports on PMN which are not anymore viable after performing NETosis.
There are many more ways to induce NETosis that not have been addressed. This precludes the general statement of the title.
The Material and method section does still not carefully describe the exact procedure (medium, supplement, precoating culture conditions and so on). This is of major importance since subtle changes in experimental procedures influence cellular responses dramatically.
All statements made in the manuscript are much too generalized. The only statement that can be made from the limited set of data and experiments is: under certain circumstances NETosis can also be observed in the absence of RIPK3-MLKL signaling. However, in other conditions the RIPK3-MLKL signaling pathway may be essential.

Reviewer: 3
Comments to the Author
The authors have not addressed my comments and the same technical problems persist. For example:

In Fig. 1D NET release in the presence of "activators" is only twice as high as the medium. Is this significant? In my opinion it is not. The images shown in Fig. 1C show very few NETs that are still difficult to quantitate and mostly intact neutrophils. The naive unactivated controls are missing. It is unclear to me how the data in Fig. 1B showing 40% of NETing cells are obtained form these images in 1C.

Figure 2A, How can anyone count the number of NETing neutrophils from the panels ont he right? The authors stated in their response that they "count only those n
eutrophils actively involved in DNA release." It is impossible for me to decipher this from these images. The deformed NET structures could originate from a far away cell and spread across a wide area. Only undisturbed unfixed "native" NETs can be counted. And counting by eye is very subjective.

How do the E. coli images look?

The authors selectively use the literature. For example, the "elegant" Science paper Brinkmann et al. was hampered by the use of Ficoll-purified cells and the absence of naive unactivated controls. Ficoll purification preactivated cells and many neutrophils released NETs during plating and prior to PMA activation. Hence the mistaken conclusion that NETs were released within 5 minutes. The Zychlinsky lab has since clarrified this issue in subsequent highly-cited papers which the authors do not take into consideration. PMA and C. albicans induce NETs in 2-4 hrs and not within 5 minutes.

What is the difference between NETosis and NET formation???

I still think that the title does not reflect the main message of the paper which could be paraphrased as "NET release does not originate form dying cells".

Authors' appeal – 20 July 2015

Dear Prof. McInnes, dear colleagues,

Thank you for your E-mail message of July 17, 2015. Since we do not agree with the reviewers' point of view and consequently with the decision, we would like to respond hoping that you will be able to reconsider our manuscript for publication in European Journal of Immunology. The language of the external reviewers has been quite unfa-vorable. Since you invited us to send a revised version, we understood that you did not necessarily share the view of the external reviewers and wanted to give us a sec-ond chance. Moreover, we understood that your concern was that the review process be fair (see also the Editorial of A. Radbruch entitled “Fair play at EJI”). We feel that in our case the process of peer review has not been fair.

From the tone of the external reviewers' comments, it is clear that no revision will ever be able to satisfy them. We did our best to reply to criticisms that included addi-tional experimentation and responded in a polite way. In such a situation, we would wish that the responsible Associate Editor review the manuscript

himself. From your letter, it seems that the decision was made based on the view of external reviewers only. Some of the criticism is simply incorrect (for example, already in the first com-ment of Reviewer 2; one can easily identify the “40% of NETing cells” in Fig. 1B) and such errors can easily be recognized by an Associate Editor.

The main problem of the external reviewers is that they are committed to a dogma whereby they see NET formation as a consequence of neutrophil death, often called “NETosis”. However, our laboratories experience cannot support this view. We clear-ly see NET formation in the absence of cell death. Therefore, we believe the term “NETosis” to be a misnomer; hence, we use the term “NET formation”.

Reviewer 3 appears to have personal insight into the work of Zychlinky’s laboratory (we explicitly asked to exclude reviewers who have worked in this lab). This reviewer seems to accept that NETs are released during plating and prior to PMA activation. Even if one would assume that the activation of neutrophils occurs under these circumstances in a nonspecific way, it is an activation process in the absence of cell death. Therefore, this reviewer ought to accept that NET formation does not require cell death. The other problem is the unpublished information that this reviewer seems to have regarding the Zychlinsky paper. We are not aware that Zychlinsky has ever published a correction of his Science (2004) paper. In the absence of a correction, we are surely justified in using the findings of his paper for our argumentation as we have done in our earlier point-by-point reply.

We are convinced that our data are entirely correct and deserve publication. We also cannot see any argument of the reviewers that would indicate that the take-home message of our work is wrong. In fact, the reviewers even share our view that the topic is important and interesting. Therefore, we would appreciate your going over our earlier and current responses to the reviewers and forming your own opinion.

Response to Reviewer 2
In their manuscript "The formation of neutrophil extracellular traps does not depend on the RIPK3-MLKL signaling pathway” Amini and colleagues investigated the role of the necroptosis pathway in the formation of extracellular traps. In face of the importance of NETosis the topic is of extremely high importance, if the analyses are performed in a proper way.
The manuscript is still rather incomplete.
This is a brief report; we could add more supplementary materials.

The general statement of the title is still an overinterpretation of a very limited amount of in vitro experi-ments.
The key point that we would like to deliver is that RIPK3 KO and WT mouse neutrophils form NETs in the same way upon activation, not requiring necroptosis.

European Journal of Immunology

There is no doubt that there are reports on PMN, which are not anymore viable after performing NETosis. PMA does kill neutrophils within a few hours. However, NET formation does not require cell death and occurs within minutes. Therefore, we believe that the term "NETosis" is inaccurate since it implies a requirement for cell death in NET formation.

There are many more ways to induce NETosis that not have been addressed. This precludes the general statement of the title.

The purpose of the article is to contribute to our understanding of NET formation and not "NETosis".

The Material and method section does still not carefully describe the exact procedure (medium, supplement, precoating culture conditions and so on). This is of major importance since subtle changes in experimental procedures influence cellular responses dramatically.

We could add more Material & Methods in the Supplementary information if requested.

All statements made in the manuscript are much too generalized. The only statement that can be made from the limited set of data and experiments is: under certain circumstances NETosis can also be observed in the absence of RIPK3-MLKL signaling. However, in other conditions the RIPK3-MLKL signaling pathway may be essential.

We would like to avoid the NETosis terminology. We focus in our investigations on what is required for NET formation.

Response to Reviewer 3

The authors have not addressed my comments and the same technical problems persist. For example: In Fig. 1D NET release in the presence of "activators" is only twice as high as the medium. Is this significant? In my opinion it is not. The images shown in Fig. 1C show very few NETs that are still difficult to quantitate and mostly intact neutrophils. The naive unactivated controls are missing. It is unclear to me how the data in Fig. 1B showing 40% of NETing cells are obtained form these images in 1C.

We feel that our findings are being unjustly hampered, and the significance of our report is ignored by pick-ing on methodological points that does not fit "the standard experimental set up". The methods we use represent very common protocols and have been used for decades in our laboratory as well as by other major labs working with granulocytes.

In Fig. 1D, NET release in the presence of "activators" are significant (P values range between 0.0096 - 0.0003). However, this is not the main message of this figure. The main message here is that that there is no difference between WT and RIPK3 KO neutrophils. Clearly, we could add the statistics regarding the effect of the "activators". The reading is done by a fluorescence plate reader and data reported as arbitrary units. Please note that the values we obtained, including the reading for inactivated neutrophils compared to activated neutrophils, are similar to all the other recently published reports (as an example, please see Fig. 5 in Ref # 14).

European Journal of Immunology

We believe that each reader can easily appreciate that the data in Fig. 1C correlate with the data shown in Fig. 1B. The quantification is carefully done using confocal microscopy and z-stacks. Naive unactivated controls can be easily added to Fig. 1C. Quantitative data are already provided in Fig. 1B.

Figure 2A, How can anyone count the number of NETing neutrophils from the panels on the right? The authors stated in their response that they "count only those neutrophils actively involved in DNA release." It is impossible for me to decipher this from these images. The deformed NET structures could originate from a far away cell and spread across a wide area. Only undisturbed unfixed "native" NETs can be counted. And counting by eye is very subjective.

We have explained in our previous reply to the reviewers that our microscopy count is done using MitoSOX and provided them images. We could add our MitoSOX images in the Supplementary Figures. In addition, we quantified the DNA release by fluorescence plate reader (routinely used in papers reporting NET for-mation). The automated values we obtained from the plate reader actually support our counting performed by confocal microscopy. We believe that the 2 techniques used provide excellent information regarding both the biological process and the quantity of the DNA released.

How do the E. coli images look?

We can provide this image.

The authors selectively use the literature. For example, the "elegant" Science paper Brinkmann et al. was hampered by the use of Ficoll-purified cells and the absence of naive unactivated controls. Ficoll purification preactivated cells and many neutrophils released NETs during plating and prior to PMA activation. Hence the mistaken conclusion that NETs were released within 5 minutes. The Zychlinsky lab has since clarrified this issue in subsequent highly-cited papers which the authors do not take into consideration. PMA and C. albicans induce NETs in 2-4 hrs and not within 5 minutes.

The reviewer persistently ignores the fact that the mouse neutrophils derived from WT and RIP3K KO mice, were isolated completely without Ficoll purification. Bone marrow derived mouse neutrophils were isolated by negative selection using the EasySep mouse neutrophil enrichment kit (StemCell, Grenoble, France). This point was mentioned in our first reply to the reviewers and also indicated in the Methods section.

It is not appropriate to discredit the previously published paper of the Zychlinsky lab and speak on their behalf of not using an accurate method for neutrophil isolation. If this were the case, we would expect that they publish a correction. In Zychlinsky's 2004 Science paper, authors used dextran-Ficoll referring to the following paper: Y. Weinrauch, et al., Nature 417, 91 (2002). In this paper, neutrophils were isolated by sedimentation in Ficoll-Paque containing dextran T-500 (Pharmacia). It is Important to know that nowadays the Dextran T-500 is no longer added for the Ficoll-Hypaque isolation method.

The “subsequent highly-cited papers” that the reviewer might be referring to, namely Fuchs M, JCB 2007, for first time defining NETosis, included a citation to the following paper: Aga E, et al., JI 2002. These authors used Histopaque 1119 (Sigma-Aldrich). Histopaque1119 consists of polysucrose, 60 g/L, and sodium diatrizoate, 167 g/L with endotoxin (<0.3 EU/mL). According to the Methods section, the isolation procedure took about 2 hours and cells were kept at room temperature.
In our laboratory, we use the Ficoll-Hypaque method (without Dextran T-500), which is technically the same type of reagent as Histopaque 1119, containing a polysaccharide molecule and sodium diatrizoate, with a much lower concentration of endotoxin (< 0.12 EU/ml). Our isolation method requires 30 min, and cells are kept on ice the entire time to avoid unspecific activation.
For the last 25 years or so, our lab has been isolating granulocytes and performing apoptosis / necrosis / autophagy assessments as well as many other experiments requiring healthy intact neutrophils. As mentioned in our previous reply, we have tested several methods of neutrophil isolation and decided that the Ficoll-Hypaque method is the most rapid and least damaging method for viability as well as base-line activation state of the cells. We routinely test the isolated neutrophils regarding purity and viability.

What is the difference between NETosis and NET formation???
The term NETosis is used by some authors to describe a type of neutrophil cell death required for NET formation. Since we do not see cell death in association with NET formation, we avoid the term “NETosis”. We do not exclude the possibility that nuclear DNA is released following necrotic cell death. However, our data show that NET formation can occur independent of cell death.

I still think that the title does not reflect the main message of the paper, which could be paraphrased, as "NET release does not originate form dying cells".
We could modify the title.

Third Editorial Decision – 14 September 2015

Dear Prof. Simon,

Thank you for your patience while we did a deeper analysis and sought additional advice on how to proceed with your manuscript ID eji.201545615.R1 entitled "The formation of neutrophil extracellular traps does not depend on the RIPK3-MLKL signaling pathway," which was submitted to the European Journal of Immunology. The original comments of the referees can be found below.

The Editor-in-Chief, on reading the referees' reports and your appeal letter, does feel that some of the requests made by the referees are demanding and we would like to see a revised version of your manuscript which addresses our specific points as detailed below and which you offered in your appeal

letter of 20 July 2015 (attached). Given that we have received another submission on the same topic we would like to publish your papers together as companion articles, and we would be grateful if you could attend to the next revision with some urgency - thank you for understanding!

You should also pay close attention to the editorial comments included below. *In particular, please edit your figure legends to follow Journal standards as outlined in the editorial comments. Failure to do this will result in delays in the re-review process.*

Once again, thank you for submitting your manuscript to European Journal of Immunology. We look forward to receiving your revision.

Yours sincerely,
Karen Chu

on behalf of Prof. Iain McInnes

Dr. Karen Chu
Editorial Office
European Journal of Immunology
e-mail: ejied@wiley.com
www.eji-journal.eu

****

NOTE - these comments are from the original 2nd review of the paper and as such for reference and record only. Please refer to the Editorial Comments below for guidance on how to proceed with a revision.

Reviewer: 2
Comments to the Author
In their manuscript "The formation of neutrophil extracellular traps does not depend on the RIPK3-MLKL signaling pathway" Amini and colleagues investigated the role of the necroptosis pathway in the formation of extracellular traps. In face of the importance of NETosis the topic is of extremely high importance, if the analyses are performed in a proper way.
The manuscript is still rather incomplete.
The general statement of the title is still an overinterpretation of a very limited amount of in vitro experiments.
There is no doubt that there are reports on PMN which are not anymore viable after performing NETosis.

European Journal of Immunology

There are many more ways to induce NETosis that not have been addressed. This precludes the general statement of the title.

The Material and method section does still not carefully describe the exact procedure (medium, supplement, precoating culture conditions and so on). This is of major importance since subtle changes in experimental procedures influence cellular responses dramatically.

All statements made in the manuscript are much too generalized. The only statement that can be made from the limited set of data and experiments is: under certain circumstances NETosis can also be observed in the absence of RIPK3-MLKL signaling. However, in other conditions the RIPK3-MLKL signaling pathway may be essential.

Reviewer: 3

Comments to the Author

The authors have not addressed my comments and the same technical problems persist. For example:

In Fig. 1D NET release in the presence of "activators" is only twice as high as the medium. Is this significant? In my opinion it is not. The images shown in Fig. 1C show very few NETs that are still difficult to quantitate and mostly intact neutrophils. The naive unactivated controls are missing. It is unclear to me how the data in Fig. 1B showing 40% of NETing cells are obtained form these images in 1C.

Figure 2A, How can anyone count the number of NETing neutrophils from the panels ont he right? The authors stated in their response that they "count only those n
eutrophils actively involved in DNA release." It is impossible for me to decipher this from these images. The deformed NET structures could originate from a far away cell and spread across a wide area. Only undisturbed unfixed "native" NETs can be counted. And counting by eye is very subjective.

How do the E. coli images look?

The authors selectively use the literature. For example, the "elegant" Science paper Brinkmann et al. was hampered by the use of Ficoll-purified cells and the absence of naive unactivated controls. Ficoll purification preactivated cells and many neutrophils released NETs during plating and prior to PMA activation. Hence the mistaken conclusion that NETs were released within 5 minutes. The Zychlinsky lab has since clarrified this issue in subsequent highly-cited papers which the authors do not take into consideration. PMA and C. albicans induce NETs in 2-4 hrs and not within 5 minutes.

What is the difference between NETosis and NET formation???

I still think that the title does not reflect the main message of the paper which could be paraphrased as "NET release does not originate form dying cells".

EDITORIAL COMMENTS FOR SECOND REVISION OF eji.201545615R2:

Abstract:
No mention that RIPK3 is associated with necro(pto)sis - this needs to be done

Results:
The first 2 sentences, linking RIPK3 signaling with necro(pto)sis and presenting the question of whether this death is NETosis and whether it is tied to NET formation, should be in the introduction with proper citations (and not reviews, as is the case here). However the authors fail to show that loss of RIPK3 also leads to loss of cell death (not in this figure) and not in a way that the referees can align with what has been shown for NETosis (microscopy with morphology, calcein blue staining loss, Annexin V staining). Therefore the question is not answered. The authors show that RIPK3 is not required for NET formation. The question is therefore not directly answered.

No seminal papers are cited. A review [7] is cited for the association between NET formation and NETosis. A review [6] is cited to cover RIPK3 signaling is necessary for TNF-induced necrosis. The seminal paper Zychlinsky and Brinkmann, JCB 2007, should be cited for the Net/Netosis comment, and I have found 3 papers for the RIPK3-death comment below.

The authors rely heavily on the fact that in the seminal papers showing RIPK3 signaling is necessary for necrosis (Cho, Y et al. 2009 Cell; He, S et al. 2009 Cell, Zhang, D.W. et al Science 2009), necrosis is shown as "cell loss/survival/viability" in bar graphs, with measures being described as EtBr incorporation. These data are polarizing.

REVISIONS TO BE PERFORMED FOR FURTHER EVALUATION:

The authors have to mention the RIPK3-death connection in their abstract.

The cell death assays need to be shown for Fig 1 as well as for Fig 2.

The authors need to provide all the data they offered in response to Referee 3, in the rebuttal.

The authors need to cite seminal papers with the actual measurements and not reviews:

Novel cell death program leads to neutrophil extracellular traps.
Fuchs TA, Abed U, Goosmann C, Hurwitz R, Schulze I, Wahn V, Weinrauch Y, Brinkmann V, Zychlinsky A.
J Cell Biol. 2007 Jan 15;176(2):231-41.

Receptor interacting protein kinase-3 determines cellular necrotic response to TNF-alpha.
He S, Wang L, Miao L, Wang T, Du F, Zhao L, Wang X.
Cell. 2009 Jun 12;137(6):1100-11. doi: 10.1016/j.cell.2009.05.021.

RIP3, an energy metabolism regulator that switches TNF-induced cell death from apoptosis to necrosis.
Zhang DW, Shao J, Lin J, Zhang N, Lu BJ, Lin SC, Dong MQ, Han J.
Science. 2009 Jul 17;325(5938):332-6. doi: 10.1126/science.

Phosphorylation-driven assembly of the RIP1-RIP3 complex regulates programmed necrosis and virus-induced inflammation.
Cho YS, Challa S, Moquin D, Genga R, Ray TD, Guildford M, Chan FK.
Cell. 2009 Jun 12;137(6):1112-23. doi: 10.1016/j.cell.2009.05.037.

Second revision – authors' responses – 5 October 2015

Reply to EDITORIAL COMMENTS and REVIEWER 3:

Abstract:
No mention that RIPK3 is associated with necro(pto)sis - this needs to be done
We have now mentioned the association of necroptosis with RIPK3 in the abstract.

Results:
The first 2 sentences, linking RIPK3 signaling with necro(pto)sis and presenting the question of whether this death is NETosis and whether it is tied to NET formation, should be in the introduction with proper citations (and not reviews, as is the case here).

We have followed the editorial suggestions and modified the text accordingly (please see page 3, second paragraph).

However the authors fail to show that loss of RIPK3 also leads to loss of cell death (not in this figure) and not in a way that the referees can align with what has been shown for NETosis (microscopy with

European Journal of Immunology

morphology, calcein blue staining loss, Annexin V staining). Therefore the question is not answered. The authors show that RIPK3 is not required for NET formation. The question is therefore not directly answered.

We have included viability data demonstrating that Ripk3-/- mouse neutrophils are less susceptible to necroptosis (Supporting Information Fig. 1B). On the other hand, Ripk3-/- mouse neutrophils showed no increased spontaneous cell death (which is known to be apoptosis) as compared to wild-type neutrophils. In addition, we demonstrate that the stimuli used for NET formation had no effect on cell viability in either wild-type or Ripk3-/- neutrophils during NET formation (Supporting Information Fig. 1C and Movie 1).

No seminal papers are cited. A review [7] is cited for the association between NET formation and NETosis. A review [6] is cited to cover RIPK3 signaling is necessary for TNF-induced necrosis. The seminal paper Zychlinsky and Brinkmann, JCB 2007, should be cited for the Net/Netosis comment, and I have found 3 papers for the RIPK3-death comment below.

We have included the references recommended by the Editor in our manuscript (new references 7-12).

The authors rely heavily on the fact that in the seminal papers showing RIPK3 signaling is necessary for necrosis (Cho, Y et al. 2009 Cell; He, S et al. 2009 Cell, Zhang, D.W. et al Science 2009), necrosis is shown as "cell loss/survival/viability" in bar graphs, with measures being described as EtBr incorporation. These data are polarizing.

An ethidium bromide (EtBr) dye-exclusion assay was used to quantify percentage of necrotic cells that have lost cell membrane integrity. Similar to propidium iodide (PI) exclusion (used in one of your recommended papers (Cho YS, Challa S, Moquin D, Genga R, Ray TD, Guildford M, Chan FK. Cell. 2009)), EtBr is also a DNA intercalating fluorescent dye and cell impermeable. Only in case of a damaged plasma membrane, as observed in primary or secondary necrotic cells, is EtBr able to enter the cell.

REVISIONS TO BE PERFORMED FOR FURTHER EVALUATION:

The authors have to mention the RIPK3-death connection in their abstract.
We have now mentioned the association of necroptosis with RIPK3 in the abstract.

The cell death assays need to be shown for Fig 1 as well as for Fig 2.
Data on cell death are now added for Figure 1 in Supporting Information Fig. 1B and 1C as well as Movie 1, and for Figure 2 in Supporting Information Fig. 2.

The authors need to provide all the data they offered in response to Referee 3, in the rebuttal

All data we offered in our rebuttal letter are now included in the new revised manuscript.

The authors need to cite seminal papers with the actual measurements and not reviews:
Novel cell death program leads to neutrophil extracellular traps. Fuchs TA, Abed U, Goosmann C, Hurwitz R, Schulze I, Wahn V, Weinrauch Y, Brinkmann V, Zychlinsky A. J Cell Biol. 2007 Jan 15;176(2):231-41.
Receptor interacting protein kinase-3 determines cellular necrotic response to TNF-alpha. He S, Wang L, Miao L, Wang T, Du F, Zhao L, Wang X. Cell. 2009 Jun 12;137(6):1100-11. doi: 10.1016/j.cell.2009.05.021.
RIP3, an energy metabolism regulator that switches TNF-induced cell death from apoptosis to necrosis. Zhang DW, Shao J, Lin J, Zhang N, Lu BJ, Lin SC, Dong MQ, Han J. Science. 2009 Jul 17;325(5938):332-6. doi: 10.1126/science.
Phosphorylation-driven assembly of the RIP1-RIP3 complex regulates programmed necrosis and virus-induced inflammation. Cho YS, Challa S, Moquin D, Genga R, Ray TD, Guildford M, Chan FK. Cell. 2009 Jun 12;137(6):1112-23. doi: 10.1016/j.cell.2009.05.037.

We have now included the above listed references in our manuscript.

_ _ _ _ _ _ _ _ _ _

Reviewer: 3
Comments to the Author
The authors have not addressed my comments and the same technical problems persist. For example:
In Fig. 1D NET release in the presence of "activators" is only twice as high as the medium. Is this significant? In my opinion it is not. The images shown in Fig. 1C show very few NETs that are still difficult to quantitate and mostly intact neutrophils. The naive unactivated controls are missing. It is unclear to me how the data in Fig. 1B showing 40% of NETing cells are obtained form these images in 1C.

Our reply in Rebuttal letter: We feel that our findings are being unjustly hampered, and the significance of our report is ignored by picking on methodological points that do not fit “the standard experimental set up”. The methods we use represent very common protocols and have been used for decades in our laboratory as well as by other major labs working with granulocytes.
In Fig. 1D, NET release in the presence of “activators” are significant (P values range between 0.0096 - 0.0003). However, this is not the main message of this figure. The main message here is that that there is no difference between WT and RIPK3 KO neutrophils. Clearly, we could add the statistics regarding the effect of the “activators”. The reading is done by a fluorescence plate reader and data reported as arbitrary units. Please note that the values we obtained, including the reading for inactivated neutrophils compared to activated neutrophils, are similar to all the other recently published reports (as an example, please see Fig. 5 in Ref # 9 (new ref. 15)).

European Journal of Immunology

We believe that each reader can easily appreciate that the data in Fig. 1C correlate with the data shown in Fig. 1B. The quantification is carefully done using confocal microscopy and z-stacks. Naive unactivated controls can be easily added to Fig. 1C. Quantitative data are already provided in Fig. 1B.

We have now added the statistical significance between control and activated neutrophils in all panels. We have included the naive unactivated control pictures (medium) for wild-type and RIPk3-deficient mouse neutrophils (Fig. 1C) as well as for control and NSA-treated human neutrophils (Fig. 2A).

Figure 2A, How can anyone count the number of NETing neutrophils from the panels on the right? The authors stated in their response that they "count only those neutrophils actively involved in DNA release." It is impossible for me to decipher this from these images. The deformed NET structures could originate from a far away cell and spread across a wide area. Only undisturbed unfixed "native" NETs can be counted. And counting by eye is very subjective.

Our reply in Rebuttal letter: We have explained in our previous reply to the reviewers that our microscopy count is done using MitoSOX and provided them images. We could add our MitoSOX Red images in the Supplementary Figures. In addition, we quantified the DNA release by fluorescence plate reader (routinely used in papers reporting NET formation). The automated values we obtained from the plate reader actually support our counting performed by confocal microscopy. We believe that the 2 techniques used provide excellent information regarding both the biological process and the quantity of the DNA released.

For quantification of released DNA, we routinely stain the cells with MitoSOX Red and Hoechst 33342. Using this type of staining, we have the highest detection sensitivity for released DNA, and at the same time we can observe the morphology of the nuclei. We now added these pictures in Supporting Information Fig. 1A (mouse neutrophils). For human neutrophils, we have already shown representative images in previously submitted versions of our work (Fig. 2A, right panel). In addition, to appreciate the three-dimensional aspect of NET formation, we provided a z-stack confocal image presented as a movie that also shows no evidence of cell death during DNA release (Supporting Information Movie 1.

How do the E. coli images look?

Our reply in Rebuttal letter: We can provide this image.

Please see Supporting Information Fig. 1A , E. coli-GFP (yellow) being entrapped by NETs.

The authors selectively use the literature. For example, the "elegant" Science paper Brinkmann et al. was hampered by the use of Ficoll-purified cells and the absence of naive unactivated controls. Ficoll purification preactivated cells and many neutrophils released NETs during plating and prior to PMA activation.

European Journal of Immunology

Hence the mistaken conclusion that NETs were released within 5 minutes. The Zychlinsky lab has since clarrified this issue in subsequent highly-cited papers which the authors do not take into consideration. PMA and C. albicans induce NETs in 2-4 hrs and not within 5 minutes.

Our reply in Rebuttal letter: The reviewer persistently ignores the fact that the mouse neutrophils derived from WT and RIP3K KO mice, were isolated completely without Ficoll purification. Bone marrow derived mouse neutrophils were isolated by negative selection using the EasySep mouse neutrophil enrichment kit (StemCell, Grenoble, France). This point was mentioned in our first reply to the reviewers and also indicated in the Methods section.

It is not appropriate to discredit the previously published paper of the Zychlinsky lab and speak on their behalf of not using an accurate method for neutrophil isolation. If this were the case, we would expect that they publish a correction. In Zychlinsky's 2004 Science paper, authors used dextran-Ficoll referring to the following paper: Y. Weinrauch, et al., Nature 417,91 (2002). In this paper, neutrophils were isolated by sedimentation in Ficoll-Paque containing dextran T-500 (Pharmacia). It is Important to know that nowadays the Dextran T-500 is no longer added for the Ficoll-Hypaque isolation method.

The "subsequent highly-cited papers" that the reviewer might be referring to, namely Fuchs M, JCB 2007, for first time defining NETosis, included a citation to the following paper: Aga E, et al., JI 2002. These authors used Histopaque 1119 (Sigma-Aldrich). Histopaque1119 consists of polysucrose, 60 g/L, and sodium diatrizoate, 167 g/L with endotoxin (<0.3 EU/mL). According to the Methods section, the isolation procedure took about 2 hours and cells were kept at room temperature.

In our laboratory, we use the Ficoll-Hypaque method (without Dextran T-500), which is technically the same type of reagent as Histopaque 1119, containing a polysaccharide molecule and sodium diatrizoate, with a much lower concentration of endotoxin (< 0.12 EU/ml). Our isolation method requires 30 min, and cells are kept on ice the entire time to avoid unspecific activation.

For the last 25 years or so, our lab has been isolating granulocytes and performing apoptosis / necrosis / autophagy assessments as well as many other experiments requiring healthy intact neutrophils. As mentioned in our previous reply, we have tested several methods of neutrophil isolation and decided that the Ficoll-Hypaque method is the most rapid and least damaging method for viability as well as baseline activation state of the cells. We routinely test the isolated neutrophils regarding purity and viability.

We have provided viability data that demonstrate that mouse and human neutrophils are more than 98% (human neutrophils) and 95% (mouse neutrophils), respectively, viable after NET formation (<1 h of activation), please see Supporting Information Fig. 1C and Fig. 2.

What is the difference between NETosis and NET formation???

Our reply in Rebuttal letter: The term NETosis is used by some authors to describe a type of neutrophil cell death required for NET formation. Since we do not see cell death in association with NET formation,

we avoid the term “NETosis”. We do not exclude the possibility that nuclear DNA is released following necrotic cell death. However, our data show that NET formation can occur independent of cell death.

I still think that the title does not reflect the main message of the paper, which could be paraphrased as "NET release does not originate form dying cells".

Our reply in Rebuttal letter: We could modify the title.

We have modified the title of the manuscript as follows: “NET formation is independent of necroptosis”. Alternatively, we would agree to the following title that would be more close to the one suggested by Reviewer 3: “NET formation does not originate with necroptotic neutrophils”.

Fourth Editorial Decision – 29 October 2015

Dear Prof. Simon, Dr. Yousefi,

It is a pleasure to provisionally accept your manuscript entitled "NET formation is independent of necroptosis" for publication in the European Journal of Immunology. For final acceptance, please follow the instructions below and return the requested items as soon as possible as we cannot process your manuscript further until all items listed below are dealt with.

Please note that EJI articles are now published online a few days after final acceptance (see Accepted Articles: http://onlinelibrary.wiley.com/journal/10.1002/(ISSN)1521-4141/accepted). The files used for the Accepted Articles are the final files and information supplied by you in Manuscript Central. You should therefore check that all the information (including author names) is correct as changes will NOT be permitted until the proofs stage.

We look forward to hearing from you and thank you for submitting your manuscript to the European Journal of Immunology.

Yours sincerely,
Karen Chu

on behalf of Prof. Iain McInnes

Dr. Karen Chu
Editorial Office

Peer review correspondence

European Journal of Immunology
e-mail: ejied@wiley.com
www.eji-journal.eu
